# Saline as a vehicle control does not alter ventilation in male CD‐1 mice

**DOI:** 10.14814/phy2.13702

**Published:** 2018-05-20

**Authors:** Candace N. Receno, Taylor G. Glausen, Lara R. DeRuisseau

**Affiliations:** ^1^ Department of Biological Sciences Le Moyne College Syracuse New York

**Keywords:** Arterial blood sampling, barometric plethysmography, ventilation

## Abstract

Saline (0.9% NaCl) is used in clinical and research settings as a vehicle for intravenous drug administration. While saline is a standard control in mouse studies, there are reports of hyperchloremic metabolic acidosis in high doses. It remains unknown if metabolic acidosis occurs in mice and/or if compensatory increases in breathing frequency and tidal volume accompany saline administration. It was hypothesized that saline administration alters blood pH and the pattern of breathing in conscious CD‐1 male mice exposed to air or hypoxia (10% O_2_, balanced N_2_). Unrestrained barometric plethysmography was used to quantify breathing frequency (breaths/min; bpm), tidal volume (VT; mL/breath/10 g body weight (BW)), and minute ventilation (VE; mL/min/10 g BW) in two designs: (1) 11‐week‐old mice with no saline exposure (*n* = 11) compared to mice with 7 days of 0.9% saline administration (intraperitoneal, i.p.; 10 mL/kg body mass; *n* = 6). and (2) 17‐week‐old mice tested before (PRE) and after 1 day (POST1, *n* = 6) or 7 days (POST7, *n* = 5) of saline (i.p.; 10 mL/kg body mass). There were no differences when comparing frequency, VT, or VE between groups for either design with room air or hypoxia exposures. Hypoxia increased frequency, VT, and VE compared to room air. Moreover, conscious blood sampling showed no differences in pH, p_a_ _CO__2_, p_aO2_, or ${\text{HCO}}_{3}^{-}$ in mice without or with 7 days of saline. These findings reveal no differences in ventilation following 1 and/or 7 days of saline administration in mice. Therefore, the use of 0.9% saline as a control is supported for studies evaluating the control of breathing in mice.

## Introduction

Saline (0.9% NaCl) is often implemented as a resuscitation solution and/or conduit for drug administration in the hospital and research setting (Takahara et al. 1977; Gainetdinov et al. 1999; Gaiardi et al. 2001; Waters et al. 2001; Hagiwara et al. 2008). Its original use was intended to mimic blood plasma during the cholera epidemic and the component concentrations have been continuously adjusted over the years (Awad et al. 2008). Currently, a 0.9% NaCl solution is now considered “normal” saline, with other saline concentrations (i.e., 3% and 5%) also on the market. In addition to a treatment option for trauma and surgery patients, saline is used in research for drug delivery and as a vehicle control (Takahara et al. 1977; Omigbodun et al. 1991; Vezina et al. 1999; Gaiardi et al. 2001; Chien et al. 2012).

Although there has been some question about the efficacy of 0.9% saline compared to other solutions, 0.9% saline has been shown to be better tolerated and perform similar to other mixtures in drug delivery studies (Salauze and Cave 1995; Sharma et al. 2013; Al‐bahadily et al. 2017). Hyperchloremic metabolic acidosis may be a negative consequence of 0.9% normal saline administration (Prough and Bidani 1999; Scheingraber et al. 1999; Waters et al. 2001), with delivery of larger volumes associated with higher degrees of metabolic acidosis (Barker 2015). It is suggested that this state of acidosis results from dilution of the serum bicarbonate present in blood (Hoorn 2017), and can occur from both intravenous (i.v.) administration and intraperitoneal (i.p.) injection (Turner et al. 2011). Intraperitoneal injection serves as a more feasible delivery method in the rodent model, with a 10 mL/kg dose being used commonly in the literature (Gainetdinov et al. 1999; Hosseinzadeh and Younesi 2002; Hagiwara et al. 2008; Sirisha et al. 2015; Zolfagharian et al. 2016; Wang et al. 2017). A volume of 10 mL/kg is considered the highest recommended amount for i.p., and this dose was chosen to elicit a maximal response.

During states of metabolic acidosis, increased ventilation may occur as a means of restoring blood to normal pH. While bicarbonate levels and changes in breathing are typically able to adjust small changes in pH, it is not known how chronic saline dosing may affect this homeostatic process. Since saline is often used as a conduit for drug delivery research, compensatory breathing patterns would have implications for outcome measures and experimental design. For example, alterations in venous return, cardiac output, and blood flow have been reported with hyperventilation due to modulation of autonomic control (Prysroberts et al. 1968; Jung et al. 1971; Ford et al. 1995). Moreover, it is possible that alterations in breathing may be exacerbated when implemented in the face of a challenge, such as hypoxia.

In order to quantify the breathing response to saline administration, two separate models were implemented. Mice were tested either 1 or 7 days after saline administration, to identify both short term and chronic effects. Animals were tested 12–16 h following saline delivery, to mimic time courses in drug delivery experiments (Ogier et al. 2007; Moore et al. 2012; Khemiri et al. 2016). Moreover, testing at these time points ensured that results were not compounded by handling procedures that are inherent to i.p. injection. We hypothesized that both time courses of saline administration would result in higher ventilation in the mouse model, as well as alterations in blood pH.

To reveal the effect of short term and chronic 0.9% saline administration on the response to hypoxia, we implemented a 10% O_2_ hypoxic bout. This level of hypoxic gas mixture has frequently been used in the literature to test the upper capacity of the respiratory system, as it leads to increased ventilatory parameters compared to baseline measures (Green and Kass 1965; Li et al. 2009; Savale et al. 2009). Reports in humans and goats have shown that alterations in resting pH can augment the breathing response to exercise and hypoxia (Forster and Klausen 1973; Smith et al. 1984). Specifically, metabolic acidosis led to slight increases in ventilatory parameters in a small cohort of humans when challenged with 64 h of hypoxic gas (Forster and Klausen 1973). Hence, we hypothesized that acidosis resulting from saline administration would result in an additive effect to the response with hypoxic gas.

## Methods

### Ethical approval

All procedures were approved by the Le Moyne College Institutional Animal Care and Use Committee (IACUC; Signatory: Environmental Health and Safety Director, IACUC Chair, Veterinarian; 2 different protocols approved 2013 & 2014). All use of animals was in accordance to the policies described in the Guide for the Care and Use of Laboratory Animals (Institute for Laboratory Animal Research, 2011). Investigators of this study understood the ethical principles under which *Physiological Reports* operates, and this work complies with its animal ethics.

### Animals

A total of 35 male CD‐1 mice obtained from Charles River Laboratories (Wilmington, MA) were used for two related study designs. Twenty‐nine were used for plethysmography, and an extra six mice (in addition to five mice from Model 2 animals) were used for blood sampling collection. For blood sampling measures, only four of the five Model 2 mice were successfully cannulated. Mice were housed for 2 weeks prior to testing, to allow animals to acclimate to the environment. Mice were housed under standard laboratory environmental conditions (12‐h light–dark cycle) and provided food and tap water ad libitum.

### Study design – Model 1

**Figure 1:**
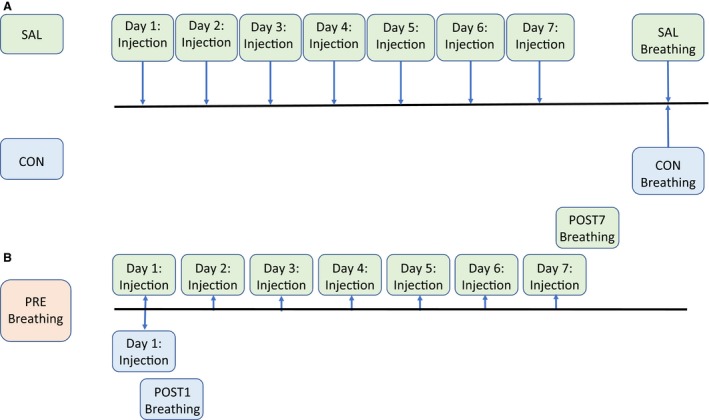
Comparison of Model 1 and Model 2 experimental design. (A) In Model 1, injections of 0.9% saline were given to 11‐week‐old male CD‐1 mice for 7 days (SAL,* n* = 7) before breathing was quantified via unrestrained barometric plethysmography (URP). A control (CON,* n* = 11) group also had breathing quantified after 7 days, but no injections were given prior to this, and (B) In Model 2, CD‐1 mice also received 0.9% saline injections. The 17‐week‐old animals were initially tested through URP (PRE,* n* = 11) and then either given 1 day (POST1, *n* = 6) or 7 days (POST7, *n* = 5) of saline before undergoing another URP trial.

This study had two different models of saline administration, as shown in Figure 1. In Model 1, a group of 11‐week‐old mice were used (body weight, BW: 34.9 (4.5) grams; Fig. 1A). One group (SAL, *n* = 7), received 7 days of daily administration of saline via intraperitoneal (i.p.) injection (10 mL/kg body mass). Breathing was quantified 12–16 h after the final i.p. injection using unrestrained barometric plethysmography (URP), as described below. The control group (CON, *n* = 11) received no prior saline administration before undergoing the URP protocol.

### Study design – Model 2

The second experimental model utilized 17‐week‐old CD‐1 (BW: 39.9 (2.8) grams) mice as their own control group (Fig. 1B). All mice underwent initial URP testing (PRE, *n* = 11) before being separated into two different groups. One group had their breathing quantified the day after the first saline i.p. injection (POST1, *n* = 6), and another had their breathing quantified after the seventh day of i.p. injections (POST7, *n* = 5).

### 0.9% Saline injections

Mice were injected with saline depending on model design. For Model 1, saline (10 mL/kg body mass) was administered to each mouse via i.p. injection for 7 days. The second model required mice to first undergo URP, before being subdivided into different groups. Groups underwent either 1 day (POST1) or 7 days (POST 7) of i.p. saline administration (10 mL/kg body mass), followed by URP.

### URP

This experiment utilized URP to obtain conscious measurements of breathing frequency (breaths/min), tidal volume (mL/breath/10 g BW), and minute ventilation (mL/min/10 g BW) in mice. Animal breathing patterns were obtained using the computer software program, Ponemah (Data Science International, DSI; St. Paul, MN). A barometric pressure, temperature, and humidity probe were fitted into the chamber and recorded offline for use in the tidal volume calculation. After a two‐point flow calibration, the mouse was put into a Buxco (DSI, St. Paul, MN, USA) URP chamber containing a reference chamber and an animal chamber; a Validyne (Validyne Engineering, Northridge, CA, USA) transducer was connected to each chamber for pressure measurements and amplified with a signal conditioner. Fresh air was pumped into the animal chamber at 0.3 L/min to maintain adequate oxygen levels. Mice were allowed to acclimate for at least 30 min, with fresh air circulated through the chamber. Between the following 30 min to 3 h, a 10‐min baseline of quiet breathing with air was recorded and used for baseline analysis in Models 1 and 2. Once baseline was acquired, defined by only minor instances of sniffing or grooming, hypoxic gas (10% O_2_; balanced N_2_) was introduced into the chamber for ten continuous minutes for Model 2 analysis. Hypoxia measures were taken to investigate whether or not a respiratory challenge would exacerbate any alterations in breathing from saline administration. The mouse was removed from the chamber and body temperature of the mouse was recorded at this time and compared to the pre‐URP temperature value. If a change in body temperature by more than 1°C was measured between pre‐ and post‐ URP values, then the difference was used in calculating hypoxia tidal volume using the Drorbaugh and Fenn equation (Drorbaugh and Fenn 1955). The assumption was made that the change in body temperature occurred during hypoxia, since this intervention is known to alter core body temperature (Yuen et al. 2012). Analysis of data was completed using Ponemah 4.0 and 5.0 (DSI, St. Paul, MN, USA).

### Baseline metabolic measures

Metabolic data were collected during the 10‐min baseline breathing period with room air (20.93% O_2_; balanced N_2_). The STPD flows into and out of the chamber were recorded (TSI Inc., Shoreview, MN, USA) throughout the experiment. A sample of URP chamber air was pulled through Nafion tubing (PermaPure LLC, Lakewood, NJ, USA) exposed to the room environment, and then through two sequential Nafion tubes placed within drierite containers (PermaPure LLC, Lakewood, NJ, USA). The dried sample went through a CD‐3A analyzer (AEI Technologies, Pittsburgh, PA, USA) for measuring the composition of CO_2_; data were collected and recorded in Ponemah for calculation of CO_2_ production. Prior to the mouse being placed in the chamber, a 10‐min average was collected for measurement of the F_i_CO_2_ of room air. The F_o_CO_2_ was the 10‐min average used during baseline collection when the mouse was in the chamber. VCO_2_ was calculated by STPD flow × (F_i_CO_2_–F_o_CO_2_).

### Cannulation surgery and blood sampling

Blood sampling was performed on a total of eleven mice, four of which were successful from the Model 2 cohort. Mice were anesthetized initially with 3% isoflurane for induction and then transitioned to 2% isoflurane for anesthetic maintenance. Throughout the procedure a toe pinch was routinely administered to confirm anesthetic depth. A central incision was made to the throat or leg and a catheter (Braintree Scientific, Braintree, MA, USA) was inserted into the carotid or femoral artery with great care to minimize damage to surrounding tissues. After patency was confirmed, the catheter was secured in place with 6‐0 suture, filled with saline, and the distal end plugged. The catheter was then fed under the skin and sutured in placed at the point of exposure on the dorsal side of the mouse. Rimadyl tablets (Zoetis, Parsippany, NJ, USA) were placed in the cage after surgery and cages were adjusted to accommodate the catheter to prevent accidental snagging and pulling of the catheter.

Blood sampling occurred after the mice had recovered in their home cage; recognized by exploring, ambulating and eating. While mice were sitting in their home cage, a tether was attached to the exposed catheter and 0.1 mL sample was withdrawn. Blood gas data were measured using iSTAT CG8+ cartridges (Abbott Point of Care Inc, Princeton, NJ, USA). A sample was taken after 7 days of saline, but within 12 h of the last injection. The control group consisted of animals with no previous injections or animals that had at least 24 h of saline washout prior to blood sampling. Once all components of the experimental design authorized by the IACUC were carried out, animals were humanely euthanized through deep anesthetizing via 4–5% isoflurane before surgical removal of the diaphragm and heart.

### Analysis

Descriptive statistics were used to obtain means and standard deviations (SD). Outcomes were analyzed using unpaired t‐tests (Model 1 and blood sampling experiments) and paired t‐tests (Model 2) with significance set a priori at *P* < 0.05 to elucidate the role of 0.9% saline administration on breathing patterns in mice during normal room air and in hypoxic exposure. Bonferroni corrections were employed to further elucidate any differences between groups. Data were only excluded if they were extreme outliers, as defined by a value ≥2SD above mean. IBM SPSS Statistics 25 (IBM, Armonk, NY, USA) and Prism 6 (GraphPad Software, La Jolla, CA, USA) were used for statistical analyses. Data are presented as mean (SD).

## Results

### Model 1

**Figure 2:**
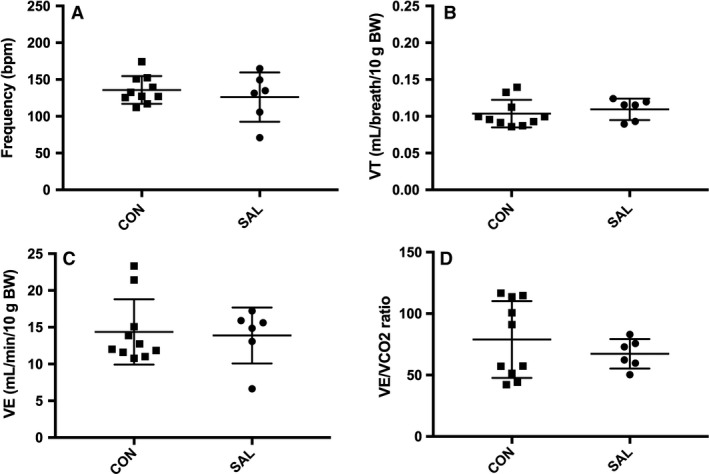
Model 1 10‐min average of ventilatory measures in room air for SAL (7‐days saline administration; *n* = 7) and CON (*n* = 11) groups. No significant differences for (A) Frequency, (B) Tidal volume (VT), (C) Minute ventilation (VE), and (D) VE/VCO2 ratio where *P *>* *0.05. Extreme outliers as defined by ≥2SD above mean were removed from data set. Values are expressed as mean ± SD.

This design provided results comparing two separate cohorts of 11‐week‐old CD‐1 male mice that were either a control (CON, *n* = 11) or given 7 days of saline administration (SAL, *n* = 7). Body weight was significantly different between CON and SAL groups at data acquisition (36.8 (1.7) vs. 31.8 (5.8) grams, respectively; *P* < 0.05), hence measures were normalized to 10 grams/body weight (10 g/BW) to account for differences in mass. This weight difference is likely due to the use of separate cohorts of mice in each group. It does not appear to be a result of the experimental intervention, since final body weight was not different from initial weight in the saline group (31.83 (5.82) vs. 31.07 (5.86), *P* > 0.05). VE was also normalized to VCO_2_ to account for possible differences in metabolism. During quiet breathing, there were no significant differences between CON and SAL groups (Fig. 2; *P* > 0.05) for frequency, VT, VE or VE/VCO_2_.

### Model 2

**Figure 3:**
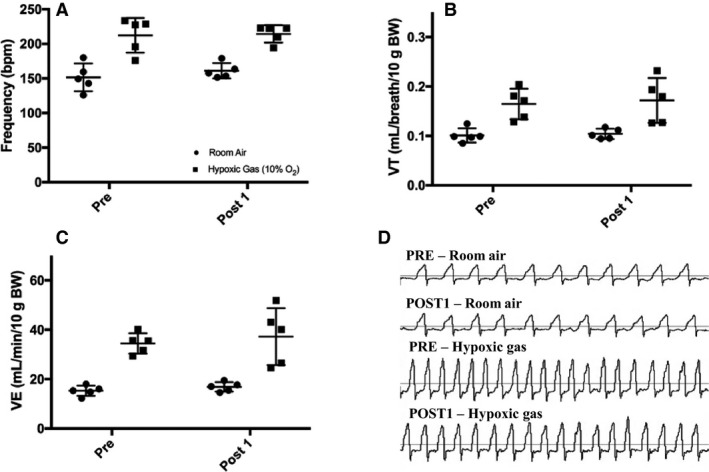
Model 2 10‐min average of ventilatory measures for PRE (*n* = 6) and POST1 (*n* = 6) groups during room air breathing and hypoxia (10% O_2_). (A) Frequency, (B) Tidal volume (VT), (C) Minute ventilation (VE), and (D) Representative flow tracings for PRE and POST1 breathing. There were no significant differences between groups (*P *>* *0.05). Extreme outlier as defined by ≥2SD above mean removed from data. Values are expressed as mean ± SD.

**Figure 4:**
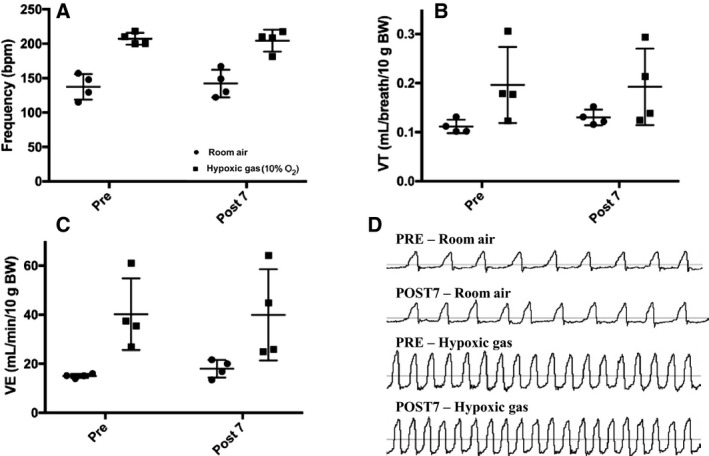
Model 2 10‐min average of ventilatory measures during room air breathing and hypoxia (10% O_2_) in PRE (*n* = 5) and POST7 (*n* = 5) groups. There were similar values reported between groups (*P *>* *0.05) for (A) Frequency, (B) Tidal volume (VT), (C) Minute ventilation (VE), and (D) Representative flow tracings for PRE and POST7 breathing. Extreme outlier as defined by ≥2SD above mean removed from data. Values are expressed as mean ± SD.

**Figure 5:**
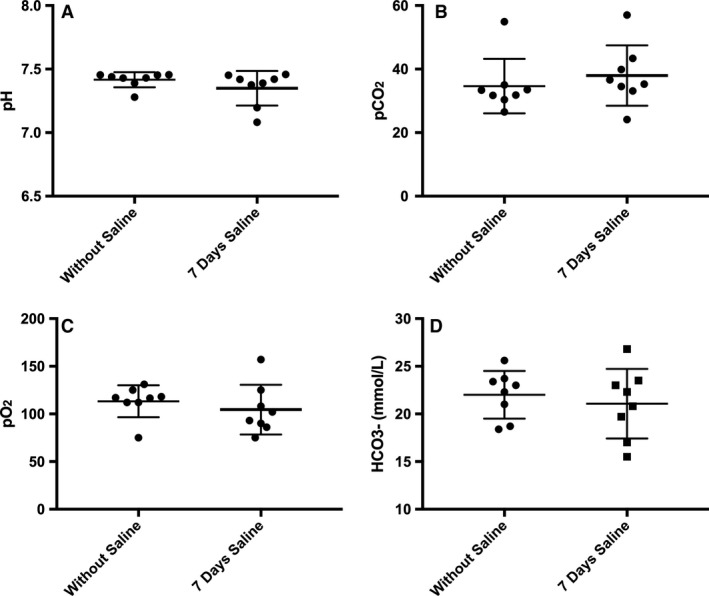
Conscious arterial blood sampling of animals. (A) pH, (B) Partial pressure of arterial CO_2_ (paCO_2_), (C) Partial pressure of arterial O_2_ (paO_2_) and (D) Bicarbonate (${\text{HCO}}_{3}^{-}$) levels within 12 h of 7 days saline administration, and without saline or following washout (>24 h after saline). No differences between saline or without saline (*n* = 8; *P *>* *0.05). Values are expressed as mean ± SD.

For Model 2, each 17‐week‐old mouse served as its own control, as a means of controlling for variability in body weight found in Model 1. Presaline weights were similar to that post saline (39.9 (2.8) vs. 39.8 (2.5) grams, respectively; *P* > 0.05). There were no differences reported during baseline with air or hypoxic (10% O_2_) response for PRE and POST1 (Fig. 3; *P* > 0.05) or PRE and POST7 (Fig. 4; *P* > 0.05) group measures which included frequency, VT, and VE. Values for PRE, POST1, and POST7 were combined into group means to observe if hypoxic challenge resulted in any changes to breathing overall. Values for frequency (150 (21) vs. 213 (17) bpm), VT (0.11 (0.02) vs. 0.18 (0.05) mL/breath/10 g BW) and VE (16.5 (2.5) vs. 37.5 (11.3) mL/min/10 g BW) within groups were significantly higher for hypoxic conditions (room air vs. hypoxic exposure, *P* < 0.001, data listed in text above), as expected. When investigating conscious blood sampling, mice with or without 7 days of saline showed no differences in pH, pCO_2_, pO_2_, or ${\text{HCO}}_{3}^{-}$ (Fig. 5).

## Discussion

The findings of this study suggest that there is no impact of 1 and/or 7 days of saline administration on patterns of breathing when mice are exposed to either room air or hypoxia. Two different models were employed to investigate ventilation after i.p. saline delivery, where Model 1 used two groups of CD‐1 mice and Model 2 tested the same cohort of mice before and after saline administration.

Previous data have indicated that saline administration may result in a state of metabolic acidosis, with dose playing a role in the degree of hyperchloremia (Prough and Bidani 1999; Scheingraber et al. 1999; Barker 2015). Ultimately, this documented state of metabolic acidosis can have large implications for breathing, as it results in a shift of the CO_2_‐ventilation curve (Van de Ven et al. 2001). Hence, gaining a better understanding of any effect of saline administration on breathing patterns is important for both research and clinical implications. For Model 1, CON and SAL groups showed similar responses to breathing frequency, VT, VE, and VE/VCO_2_ during room air breathing conditions. Moreover, PRE and POST1/POST7 groups in Model 2 also showed similar responses in normal air and hypoxic conditions. Blood pH, p_aO2_, p_aCO2_, and ${\text{HCO}}_{3}^{-}$ were not different between groups. The only significant differences were elucidated when comparing the results of normal room air breathing to hypoxic breathing, where hypoxic exposures resulted in a more pronounced breathing pattern. We also hypothesized an additive effect of saline exposure and hypoxia on breathing, but did not find differences in the hypoxic response between the PRE/POST1 or PRE/POST7 day groups. The 10% O_2_ hypoxic challenge was substantial and resulted in significant increases in breathing measures. Greater hypoxic challenges to animals (5–8% O_2_) have resulted in severe respiratory disturbances and apnea, and would not suit the purpose of this study (Guntheroth and Kawabori 1975; Han et al. 2001; Adachi et al. 2006; Zwemer et al. 2007). Therefore, it is likely the maximal (elevated) pattern of breathing response to hypoxic gas was achieved with no differences revealed by saline exposure. This is expected due to the arterial blood pH within normal physiological range, and no differences detected between saline and no saline comparisons. Ultimately, since our results do not indicate an impact of saline administration on breathing or blood pH, this suggests that saline is a useful vehicle particularly for research, as administration should not serve as a confounding factor for breathing studies.

Overall, these findings are indicative of breathing patterns several hours post saline administration. In both models, breathing was tested 12–16 h after i.p. injections, indicating a period of recuperation time between injection and testing. This was by design, as conscious breathing studies that use saline as a vehicle are often investigating a longer time course following administration. However, it is entirely possible that there are differences in breathing present immediately after administration of saline. This could be from saline itself, or the handling of the mouse, or the injection. The window for differences may be very small, as compensatory mechanisms for acidosis would occur immediately to offset this imbalance. However, we tested a time period following injection that should no longer be confounded by the needle prick or scruffing. The possible chronic effects were the main focus of our experiments.

Although the longer term (7 days) implications of saline administration were the primary emphasis of our study, we added the POST1 time point to determine if a shorter term breathing response was elicited by saline. Hence, Model 2 is able to address both a shorter term and longer term administration period. Interestingly, there were no differences compared to the PRE saline group, suggesting there are no adverse breathing effects even after 1 day of saline administration. The 7 day administration in Models 1 and 2 would be most indicative of a chronic saline effect. This timeframe would be represented in the research field where saline is often used as a vehicle for longer term experimental drug administration, or in the hospital where patients are continuously administered drugs via saline infusion.

This study used male mice in order to test the effects of saline administration on breathing. Baseline breathing measures in rodents vary between males and females, as well as across age ranges, and other differences may be further uncovered by challenges such as hypoxia (Mortola and Saiki 1996; Schulz et al. 2002; Flandre et al. 2003; Holley et al. 2012). Moreover, males and females are not uniform in their responses to environmental cues (Shanksy 2018) and these differences could lead to various responses to i.p. injections and the plethysmography chamber (Rasid et al. 2012). Importantly, mice did not become acidic in this study, which served as our primary mechanism for altered breathing patterns. While we would not expect other cohorts to become acidic with this particular saline dosing, acidosis may be more prevalent in female animals, especially in pregnancy, as contrasting basal metabolic responses can alter the degree of acidosis (Perez et al. 1980; Abel 1996). Thus, studying patterns of breathing after saline administration in other populations, such as females, pregnant dams, neonates, and older rodents, could show varied responses compared to those captured within this study and warrant further investigation.

To conclude, this was a study seeking to elucidate any potential effects of 1 or 7 days of saline administration on the pattern of breathing in male mice. Future directions would include expanding the time course and dosage to gain more data regarding breathing frequency, VT, VE, pH, p_aO2_, p_aCO2_, and ${\text{HCO}}_{3}^{-}$ following minutes and hours and/or higher concentrations of saline, as well as other populations of importance. Ultimately, these insights could aid in uncovering a better understanding of the biological impact of such a widely used solution. In conclusion, the use of two models of saline administration to mice did not reveal any changes in the patterns of breathing. These data support the continued use of saline as a vehicle for drug delivery in the research setting, as it did not result in alterations in breathing that may confound experimental designs.

## Conflict of Interest

There are no competing interests for any of the authors of this manuscript.
